# Xiaoxuming decoction cutting formula reduces LPS-stimulated inflammation in BV-2 cells by regulating miR-9-5p in microglia exosomes

**DOI:** 10.3389/fphar.2023.1183612

**Published:** 2023-05-17

**Authors:** Menglei Wang, Yuting Yang, Yanlei Guo, Ruirong Tan, Yanmei Sheng, Huawei Chui, Ping Chen, Hua Luo, Zhujun Ying, Li Li, Jin Zeng, Junning Zhao

**Affiliations:** ^1^ Translational Chinese Medicine Key Laboratory of Sichuan Province, Sichuan Academy of Chinese Medicine Sciences, Sichuan Institute for Translational Chinese Medicine, Chengdu, China; ^2^ College Pharmacy, Chengdu Medical College, Chengdu, China; ^3^ Chongqing Academy of Chinese Materia Medica, Chongqing, China; ^4^ Institute of Chinese Medical Sciences, State Key Laboratory of Quality Research in Chinese Medicine, University of Macau, Macao, China

**Keywords:** xiaoxuming decoction cutting formula, neuroinflammation, miR-9-5p, microglia exosomes, smallRNA-seq

## Abstract

**The Background:** Stroke is one of the leading causes of morbidity and mortality, and the inflammatory mechanism plays a crucial role in stroke-related brain injury and post-ischemic tissue damage. Xiaoxuming decoction (XXMD) is the first prescription for the treatment of “zhongfeng” (a broad concept referring to stroke) in the Tang and Song Dynasties of China and has a significant position in the history of stroke treatment. Through the study of ancient medical records and modern clinical evidence, it is evident that XXMD has significant efficacy in the treatment of stroke and its sequelae, and its pharmacological mechanism may be related to post-stroke inflammation. However, XXMD contains 12 medicinal herbs with complex composition, and therefore, a simplified version of XXMD, called Xiaoxuming decoction cutting (XXMD-C), was derived based on the anti-inflammatory effects of the individual herbs. Therefore, it is necessary to explore and confirm the anti-inflammatory mechanism of XXMD-C.

**Aim of the study:** Based on the previous experiments of our research group, it was found that both XXMD and XXMD-C have anti-inflammatory effects on LPS-induced microglia, and XXMD-C has a better anti-inflammatory effect. Since miRNAs in exosomes also participate in the occurrence and development of cardiovascular diseases, and traditional Chinese medicine can regulate exosomal miRNAs through intervention, this study aims to explore the anti-inflammatory mechanism of XXMD-C in the treatment of post-stroke inflammation through transcriptome sequencing, providing a basis for the application of XXMD-C.

**Materials and methods:** XXMD-C was extracted using water and filtered through a 0.22 μm membrane filter. The main chemical components of the medicinal herbs in XXMD-C were rapidly qualitatively analyzed using ultra-high-performance liquid chromatography-quadrupole time-of-flight mass spectrometry (UPLC-Q-TOF-MS). Cell viability was determined using the CCK-8 assay, and an LPS-induced BV-2 cell inflammation model was established. The expression of inflammatory cytokines was detected using ELISA and Western blot (WB). Extracellular vesicles were extracted using ultracentrifugation, and identified using transmission electron microscopy (TEM), nanoparticle tracking analysis, and WB. Differential miRNAs were screened using smallRNA-seq sequencing, and validated using RT-PCR and Western blot.

**Results:** The UPLC-Q-TOF-MS analysis revealed that representative components including ephedrine, pseudoephedrine, cinnamaldehyde, baicalin, baicalein, wogonin, and ginsenoside Rg1 were detected in XXMD-C. The results of ELISA and WB assays showed that XXMD-C had a therapeutic effect on LPS-induced inflammation in BV-2 cells. TEM, nanoparticle tracking analysis, and WB results demonstrated the successful extraction of extracellular vesicles using high-speed centrifugation. Differential miRNA analysis by smallRNA-seq identified miR-9-5p, which was validated by RT-PCR and WB. Inhibition of miR-9-5p was found to downregulate the expression of inflammatory factors including IL-1β, IL-6, iNOS, and TNF-α.

**Conclusion:** The study found that XXMD-C has anti-neuroinflammatory effects. Through smallRNA-seq sequencing of extracellular vesicles, miR-9-5p was identified as a key miRNA in the mechanism of XXMD-C for treating neuroinflammation, and its *in vivo* anti-inflammatory mechanism deserves further investigation.

## 1 Introduction

Stroke is a life-threatening cerebrovascular disease and one of the leading causes of incidence and mortality worldwide (Ahad et al., 2020). Inflammation plays a critical role in stroke-related brain injury and tissue damage following ischemia. Increasing evidence suggests that certain inflammatory mechanisms play a crucial role in the onset or acute phase of cerebral ischemia (Xie et al., 2022). The activation of microglia, which serves as the first line of defense against brain injury or disease, is the core of post-stroke inflammation (Gao et al., 2020).

In normal conditions, microglia (BV-2) are in a resting state and act as sensors to detect abnormal changes in brain damage and external stimuli. However, in the event of stroke, microglia respond by releasing inflammatory mediators, including cytokines (IL-1, IL-6, TNF-α) and chemokines (MCP-1, CXCL-1) (Xu et al., 2020). These mediators work by recruiting leukocytes from outside the brain, leukocytes enter the brain parenchyma through the blood brain barrier (BBB), aggravating the release of cytokines (Shigemoto-Mogami et al., 2018), and finally causing a series of brain injuries such as BBB destruction, neuronal damage, and vascular senescence. Ultimately, this causes a series of brain injuries, including BBB disruption, neuronal damage, and vascular aging.

Exosomes contain complex RNA and proteins, and studies have found that extracellular vesicles (EVs) secreted by neurons and glial cells (such as exosomes and cytoplasmic membrane fragments) play a crucial role in intercellular communication and neuroinflammation through mRNA, microRNA (miRNA), and proteins (Brites and Fernandes, 2015). They also participate in the damage and repair of neurons and vascular endothelial cells after stroke (Hu et al., 2019). For example, PC12 cells can secrete exosomes, which induce polarization of microglia through miR-21-5 (Rodríguez-Gómez et al., 2020)p. The miRNAs carried by exosomes can enter cells through phagocytosis and affect the cells’ response to the external environment (Tan and Han, 2015). Gui-Lou-Zi-Zhu-Tang (GLZZT) can inhibit neuroinflammation after MCAO in rats through miR-155 (Yang et al., 2021). Therefore, it is necessary to conduct in-depth research on whether microglia can secrete exosomes and transmit information through miRNAs in exosomes to affect microglial inflammatory responses.

XXMD originated from ‘Xiaopin Fang’ (Chen, 1983) and ‘Beiji Qianjin Yao Fang’ (Sun, 2009a) written by Chen Yanzi in the Eastern Jin Dynasty. Sun Simiao established the basic principle of ‘dispelling wind and supporting righteousness’ for the treatment of stroke and listed XXMD as the first prescription. It is the most important formula for the treatment of stroke in ancient China. Clinical cases of famous traditional Chinese medicine doctors (Sun, 2009b; Huang, 2011; Wang, 2011; Xiong, 2020) and studies with strong clinical evidence (Chen et al., 2010; Huang et al., 2021a; Zhang et al., 2021) have shown that XXMD is effective in treating stroke and its sequelae. Inflammation plays an important role in a series of brain damage after stroke, and XXMD’s mechanism in treating stroke may be related to inflammation. Based on our research group’s previous findings that XXMD has anti-inflammatory effects on LPS-induced BV-2 cells, we have developed a simplified version of XXMD, called XXMD-C (containing only Ma Huang, Gui Zhi, Huang Qin, and Ren Shen), by following the principle of “dispelling wind and supporting righteousness” and combining it with modern pharmacology research. The original XXMD formula contains 12 herbs with complex and difficult to control quality of individual herbal components. Moreover, XXMD has a broad range of clinical indications and cannot be specifically focused on post-stroke inflammation. In our previous studies, we evaluated the therapeutic effects of XXMD and XXMD-C on LPS-induced microglial cell inflammation. The results revealed that both formulas have anti-inflammatory effects, with XXMD-C being more effective in reducing LPS-induced microglial cell inflammation due to its simplified composition.

This study utilized UPLC-Q-TOF-MS for rapid qualitative analysis of the major chemical components in XXMD-C. A cell inflammation model was established in BV-2 cells induced by LPS, and XXMD-C was used to treat the cells to detect cell viability. The expression of inflammatory factors was detected by ELISA and Western blot. Extracellular vesicles were isolated using ultracentrifugation and identified using TEM, nanoparticle tracking analysis, and Western blot. Differential miRNAs were screened by smallRNA-seq sequencing analysis, and cell transfection was used to inhibit miRNA expression. The levels of inflammatory factors were validated using RT-PCR and Western blot to verify the mechanism.

## 2 Materials and methods

### 2.1 Drug preparation

Ephedra from New Lotus Chinese Medicine and Beverage Co. Ltd. (2111089, China). Cinnamomum from New Lotus Chinese Medicine and Beverage Co. Ltd. (2107019, China). Scutellaria from New Lotus Chinese Medicine and Beverage Co. Ltd. (2107115, China). Ginseng from New Lotus Chinese Medicine and Beverage Co. Ltd. (2112035, China). XXMD-C preparation: Ephedra, Cinnamomum, Scutellaria and Ginseng were prepared in the ratio of 1:1:1:1, one serving was calculated as 13.80 g. Add 600 mL of water, boil Ephedra “three boils” and remove the froth, then add the remaining herbs and boil 150 mL, the concentration of drug in the infusion is 368 mg/mL (original raw drug). Filtered through 0.22 μm microporous membrane and stored at −20°C in the refrigerator.

### 2.2 Chemicals and reagents

Cell Counting Kit-8 (CCK-8) from Biosharp (22082317, China). Lipopolysaccharide (LPS) from Sigma-Aldrich (039M4004V, United States). PKH26 red cell membrane staining kit from Sigma-Aldrich (MINI26-1KT, United States). High sugar Dulbecco’s Modified Eagle medium (DMEM) from Gibco (8121589, United States). Fetal bovine serum (FBS) from Gibco (s711-001s, United States). NO Assay Kit From Biyuntian Biotechnology Co. Ltd. (022421210712, China). DAPI staining solution From Biyuntian Biotechnology Co. Ltd. (C1005, China). IL-6ELISA Kit from Elabscience Biotechnology Co., Ltd. (QBEH6PJXN8, China). TNF-αELISA Kit from Elabscience Biotechnology Co., Ltd. (3JNH9CIH96, China). Molpure^®^ Cell/Tissue Total RNA Kit from YEASEN (M8223090, China). primeScript RT reagent Kit from Bao Ri Doctor Biotech (AL61920A, China). PrimeScript RT reagent kit from Bao Ri Doctor Biotech (AL61920A, China). TB Green TM Premix Ex TaqTM II (Tli RNaseH Plus) from Bao Ri Doctor Biotech (AM43092A, China). Rabbit anti-iNOS from Abcam (EPR16635, United States). Rabbit anti-IL-1β from Abcam (EPR23851-127, United States). Rabbit anti-TNF-α from Abcam (EPR19147, United States). Rabbit anti-β-Actin from Abcam (United States). Goat anti-rabbit IgG-HRP from CST (7074P2, United States).

### 2.3 UPLC/Q-TOF-MS for qualitative detection of XXMD-C components

The LC30A-UPLC (Shimadzu, Japan) uses a Kinetex XB-C18 column (100 mm × 2.1 mm, 2.6 µm) with a mobile phase consisting of ultrapure water containing 0.1% formic acid (A) and acetonitrile (B), using a gradient elution program (initial B was 10% and held for 1.00 min; B was increased to 80% from 1.00–7.00 min and held until 11.00 min; B was returned to 10% at 11.5 min and held until 15.00 min), with a flow rate of 250 μL/min and a column temperature of 30°C.

The Triple TOF 4600 (ABSCIEX, United States) high-resolution mass spectrometry system uses an ESI ion source and collects data using both Positive and Negative ionization modes. The mass scanning range is 100–1,000 m/z. The sheath gas pressure is set at 0.38 MPa, the auxiliary gas pressure is also set at 0.38 MPa, and the curtain gas pressure is set at 0.17 MPa. The ionization temperature is set at 600°C, and the system uses TOF-MS-Product Ion-IDA scanning. The first-level precursor scan and the triggered second-level Product Ion-IDA scan have ion accumulation times of 250 ms and 100 ms, respectively. The system uses multiple mass defect filtering (MMDF) and dynamic background subtraction (DBS) as the second-level triggering conditions. The declustering voltage is set at 80 V, and the collision energy (CE) is set at ±35 eV. In Positive mode, the CES collision energy is added as (35 ± 15) eV, and in Negative mode, the CES collision energy is added as (−25 ± 15) eV.

T According to the 2022 edition of the Chinese Pharmacopoeia and relevant literature, the main representative components of each medicinal herb were identified. The XIC Manager function in Peak View 1.2 software (Umetrics, Sweden) was used for preliminary screening of compounds, obtaining information such as molecular ion peaks, secondary fragment ion information, and retention time. Compounds were identified based on their accurate molecular weight combined with secondary spectrum fragment analysis.

### 2.4 Cell culture

The mouse microglial cell line BV-2 (provided by Shanghai FHS Biological Co., Ltd.) was cultured in DMEM complete medium containing 1% antibiotics (100 U/mL penicillin and 100 U/mL streptomycin) and 10% heat-inactivated exosome-free fetal bovine serum (2128190, VivaCell, China), and incubated at 37°C. and 5% CO_2_ in a cell culture incubator.

### 2.5 Cell viability assay

To evaluate the effect of XXMD-C on BV-2 cell viability, cells were seeded at a density of 1 × 10^4^ cells per well in a 96-well plate and incubated at 37°C with 5% CO_2_ for 24 h. Different concentrations of XXMD-C (10 mg/mL, 5 mg/mL, 2.5 mg/mL, and 1.25 mg/mL) were added to the wells, and the cells were further incubated for 24 h. After removing the medium, 110 μL of CCK8 solution was added to each well and incubated for 30 min. The absorbance was measured at 450 nm using a microplate reader, and the cell proliferation rate was calculated.

### 2.6 ELISA assay for NO, IL-6, and TNF-α detection

BV-2 cells were seededat a density of 2 × 10^6^ cells/well in 6-well plates with 2 mL medium per well and incubated in a 5% CO_2_, 37°C incubator for 24 h. The cells were divided into 5 groups: control, LPS, and XXMD-C high, middle, and low dose groups (5 mg/mL, 2.5 mg/mL, and 1.25 mg/mL, respectively). The LPS group was induced with 1 μg/mL LPS, and the XXMD-C groups were treated with the drug extract mixed with LPS (final concentration of 1 μg/mL LPS). The control group was not subjected to any treatment and was incubated in the incubator for 24 h. The concentrations of NO, IL-6, and TNF-α were measured in the supernatant of the cell culture using ELISA kits, and the OD values were determined at 450 nm. The cytokine release levels were calculated based on the OD values.

### 2.7 Western blot analysis

The experiment consisted of 5 groups, including the control group, LPS group (1 μg/mL LPS), low-dose group (1 μg/mL LPS + 1.25 mg/mL XXMD-C), medium-dose group (1 μg/mL LPS + 2.5 mg/mL XXMD-C), and high-dose group (1 μg/mL LPS + 5 mg/mL XXMD-C). BV-2 cells were cultured with or without XXMD-C and induced with LPS for 24 h. After washing twice with pre-cooled PBS, the cells were lysed with 150 μL RIPA lysis buffer (G2002, servicebio, China) on ice for 30 min. The lysate was collected in a 1.5 mL EP tube using a clean cell scraper and centrifuged at 13,400 g at 4°C to obtain the supernatant, and the protein concentration of each group was determined according to the instructions of the BCA kit. Proteins were denatured by adding protein loading buffer (070121211027, Beyotime, China) and boiling for 5 min at 100°C. The proteins were then separated for subsequent gel electrophoresis. The samples were subjected to 10% SDS-PAGE electrophoresis, and then transferred onto a polyvinylidene fluoride (PVDF) membrane in ice bath. The PVDF membrane was blocked with 5% skimmed milk at room temperature for 1.5 h and incubated overnight in the primary antibody solution. Finally, the PVDF membrane was incubated in the secondary antibody solution for 1 h. After washing, the protein bands were visualized using ECL solution for 1–2 min and analyzed for the gray value.

### 2.8 Extracellular vesicle isolation and identification

BV-2-Exo was isolated from the culture supernatant of BV-2 cells using ultracentrifugation. BV-2 cells were cultured in exosome-depleted DMEM for 48 h, and the cell culture supernatant was collected and centrifuged at 2000 *g* for 30 min and 10,000 ×g for 45 min at 4°C. The supernatant was filtered through a 0.45 μm membrane and ultracentrifuged at 100,000 ×g for 70 min at 4°C. The supernatant was removed, and the pellet was resuspended in 10 mL of pre-chilled 1× PBS, and then ultracentrifuged again at 100,000 ×g for 70 min at 4°C. The supernatant was removed, and the pellet was resuspended in 200 μL of pre-chilled 1× PBS and then identified. The protein content of the exosomes was measured using the BCA assay kit. Exosome markers CD63 and TSG101 were detected by Western blot, and the morphology and particle size of the exosomes were identified by transmission electron microscopy (HT-7700, Hitachi, Japan) and a nanometer particle tracking analyzer (ZetaVIEW, PARTICLE METRIX, Germany).

### 2.9 Observation of microglial uptake of extracellular vesicles using confocal laser scanning microscopy

Using red fluorescent membrane dye PKH67, BV-2-Exo was labeled. The exosomes were incubated with diluent C and PKH26 at room temperature for 5 min. PKH26-labeled exosomes were then diluted in PBS and centrifuged at 100,000 × g for 17 min at 4°C twice to remove any unincorporated dye contamination from the exosome labeling reaction. Subsequently, PKH26-labeled or denatured exosomes were co-incubated with BV-2 cells for 24 h. After cell fixation, DAPI was used for staining and observation was performed using laser confocal microscopy (sp8, Leica, Germany).

### 2.10 Small RNA sequencing analysis

Nuo he zhi yuan technology co., ltd. (Beijing, China) utilized extracellular vesicle total RNA to construct a miRNA library, which was subsequently sequenced. The total RNA samples were extracted and subjected to agarose gel electrophoresis, with 18-30 nt fragments selected for library construction. After passing the library construction quality check, the 3′ and 5′ ends of Small RNA with special structures (having a complete phosphate group at the 5′ end and a hydroxyl group at the 3′ end) were directly ligated with adapters on both ends, using total RNA as the starting sample, followed by reverse transcription to synthesize cDNA. Subsequently, the resulting cDNA library was PCR (ProFlex, Thermo, United States) amplified, and the target DNA fragments were separated using PAGE gel electrophoresis, with the gel cut and recovered to obtain the cDNA library. Quality control of the constructed library was conducted using Agilent 2,100 (Agilent Technologies, CA, United States) and PCR, followed by deep sequencing of small RNA using HiSeq™2,500/MiSeq system (Illumina Novoseq1.5).

In order to ensure the quality of information analysis, the raw reads obtained from the initial filtering were subjected to a further filtering process to remove reads with adapters and low quality, in order to obtain clean tag sequences. The clean tag sequences were then filtered to select sRNAs within a certain length range for subsequent analysis. To analyze the distribution of small RNAs on the reference sequence, the bowtie-0.12.9 (-v 0 -k 1) software was used to align sRNAs (filtered sRNAs of certain length range) to the reference sequence. The existing and known miRNAs were identified using srna-tools-cli (http://srna-tools.cm.uea.ac.uk/, --tool hp_tool), and new miRNAs were identified using miREvo_v1.1 (-i -r -M -m -k -p 10 -g 50,000) and mirdeep2_0_0_5 (quantifier.pl -p -m -r -y -g 0 -T 10) software. Next, the identified miRNAs in each sample were summarized, and the tag expression of each miRNA was calculated in million percentile, and the miRNA expression profile of all samples was obtained. The differential expression of miRNAs was analyzed using the edgeR3.2.4 (padj<0.05, |log2foldchang|>1) software, and significant and differentially expressed miRNAs were determined with log2 (fc) > 1.0 and *p* < 0.05. Additionally, volcano plots were generated using the ggplot package in R to analyze the differential expression of miRNAs, and clustering heatmaps were generated to analyze the expression patterns of miRNAs. miRNAs with tags less than one millionth were filtered out.

### 2.11 RT-PCR detection of extracellular miRNA expression

Te RNA extracted from exosomes was used for miRNA cDNA synthesis using the Prime Script RT kit. SYBR Green qPCR fluorescent detection kit was used for RT-PCR (CFX96, BIO-RAD, United States) to detect the expression of miRNAs in Te. Gene full sequences were searched from the National Center for Biotechnology Information (NCBI) database, and Primer Premier software was used to design specific primers for each gene. All primers were designed and synthesized by Shenggong Bioengineering Technology Service Co., Ltd. (Shanghai, China). RT-PCR was used for quantitative detection, and the CT (threshold cycle) values of each sample during the PCR process were analyzed. The relative mRNA expression level of X was calculated using the 2^−△△CT^ method. For each sample, three independent replicate experiments were performed. The results were expressed as mean ± standard deviation (Table 1; Table 2).

**TABLE 1:** miRNA primer design.

Primer name	Primer sequences
mmu-miR-9-5p qRT F	GCC​GAG​TCT​TTG​GTT​ATC​TAG​CT
mmu-miR-3066-5p qRT F	GCC​GAG​TTG​GTT​GCT​GTA​GAT​T
mmu-miR-128-1-5p qRT F	CGGGGCCGTAGCACT
cel-miR-39 qRT F	AGC​CCG​TCA​CCT​GGT​GTA​AAT​C
miR generic qRT R	CAG​TGC​AGG​GTC​CGA​GGT​AT

**TABLE 2:** Primer design for inflammatory factors.

Primer name	Upstream	Downstream
β-actin	cta​cct​cat​gaa​gat​cct​gac​c	cac​agc​ttc​tct​ttg​atg​tca​c
IL-1β	cac​tac​agg​ctc​cga​gat​gaa​caa​c	tgt​cgt​tgc​ttg​gtt​ctc​ctt​gta​c
IL-6	ctc​cca​aca​gac​ctg​tct​ata​c	cca​ttg​cac​aac​tct​ttt​ctc​a
iNOS	atc​ttg​gag​cga​gtt​gtg​gat​tgt​c	tag​gtg​agg​gct​tgg​ctg​agt​g
TNF-α	atg​tct​cag​cct​ctt​ctc​att​c	gct​tgt​cac​tcg​aat​ttt​gag​a

### 2.12 Cell transfection

BV-2 cells (4 × 10^5^ cells per well) were seeded onto a 6-well plate and allowed to reach 70% confluency overnight for transfection. RiboFECTTMCP transfection reagent, miR-9-5p mimic, and inhibitor were all provided by Ruibo biology technology co., ltd. (Guangzhou, China) following the manufacturer’s instructions. The transfected cells were then treated with 5 mg/mL XXMD-C at 37°C for 24 h. Subsequently, RT-PCR was performed for detection.

### 2.13 Statistical analysis

The statistical analysis and graph generation were performed using GraphPad 7.0 software. All experiments were performed in triplicate, and paired t-test and one-way analysis of variance (ANOVA) were used to determine the significance of relative expression between groups, with *p* < 0.05 considered statistically significant, *p* < 0.01 considered highly significant, and *p* < 0.001 considered extremely significant.

## 3 Results

### 3.1 Determination of the main components of XXMD-C by UPLC/Q-TOF-MS

**FIGURE 1:**
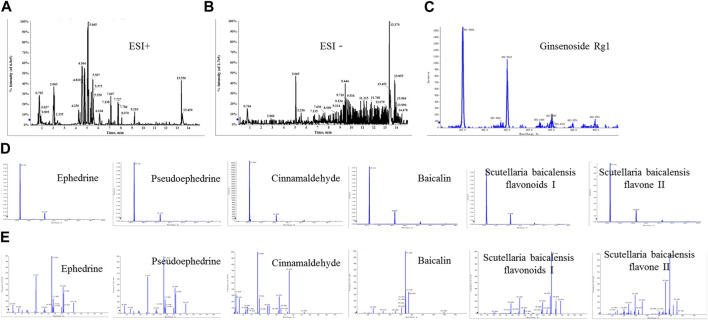
UPLC/Q-TOF-MS for the determination of the main components of XXMD-C. **(A, B)** Total ion flow diagram of XXMD-C; **(C, D)** primary mass spectra of each component of XXMD-C; **(E)** secondary mass spectra of each component of XXMD-C.

The positive and negative ion modes of the total ion chromatogram (TIC) were obtained under the above-mentioned chromatographic and mass spectrometric conditions (Figures 1A,B). The TIC of each representative component of the herbal medicines was used as representative data (Figures 1C–E). The results showed that a total of 7 chemical components were detected, including ephedrine, pseudoephedrine, ginsenoside Rg1, baicalin, baicalein, wogonoside, and cinnamaldehyde, with their accurate molecular weights and retention times listed in Table 3. The results indicated that the major components of XXMD-C prescription herbal medicines were well-characterized in the chromatogram, which could represent the overall and intrinsic quality of the XXMD-C experimental samples.

**TABLE 3:** Qualitative identification results.

Number	Representative component	Formula	tR (min)	Deviation (ppm)	Mass charge ratio (m/z)	
					Calculated mass	Measured mass
1	Ephedrine	C10H15NO	3.73	0.4	165.11536	166.1227
2	Pseudoephedrine	C10H15NO	1.97	−1	165.11536	166.1225
3	Ginsenoside Rg1	C42H72O14	6.78	1.1	800.49221	801.5004
4	Baicalin	C21H18O11	7.36	−0.9	446.08491	447.0918
5	Scutellaria baicalensis flavonoids I	C17H14O6	9.01	−0.7	314.07904	315.0861
6	Scutellaria baicalensis flavonoids I I	C19H18O8	8.91	−0.8	374.10017	375.107
7	Cinnamaldehyde	C9H8O	0.86		132.05751	133.0607

### 3.2 The effects of XXMD-C on BV-2 cell viability

**FIGURE 2:**
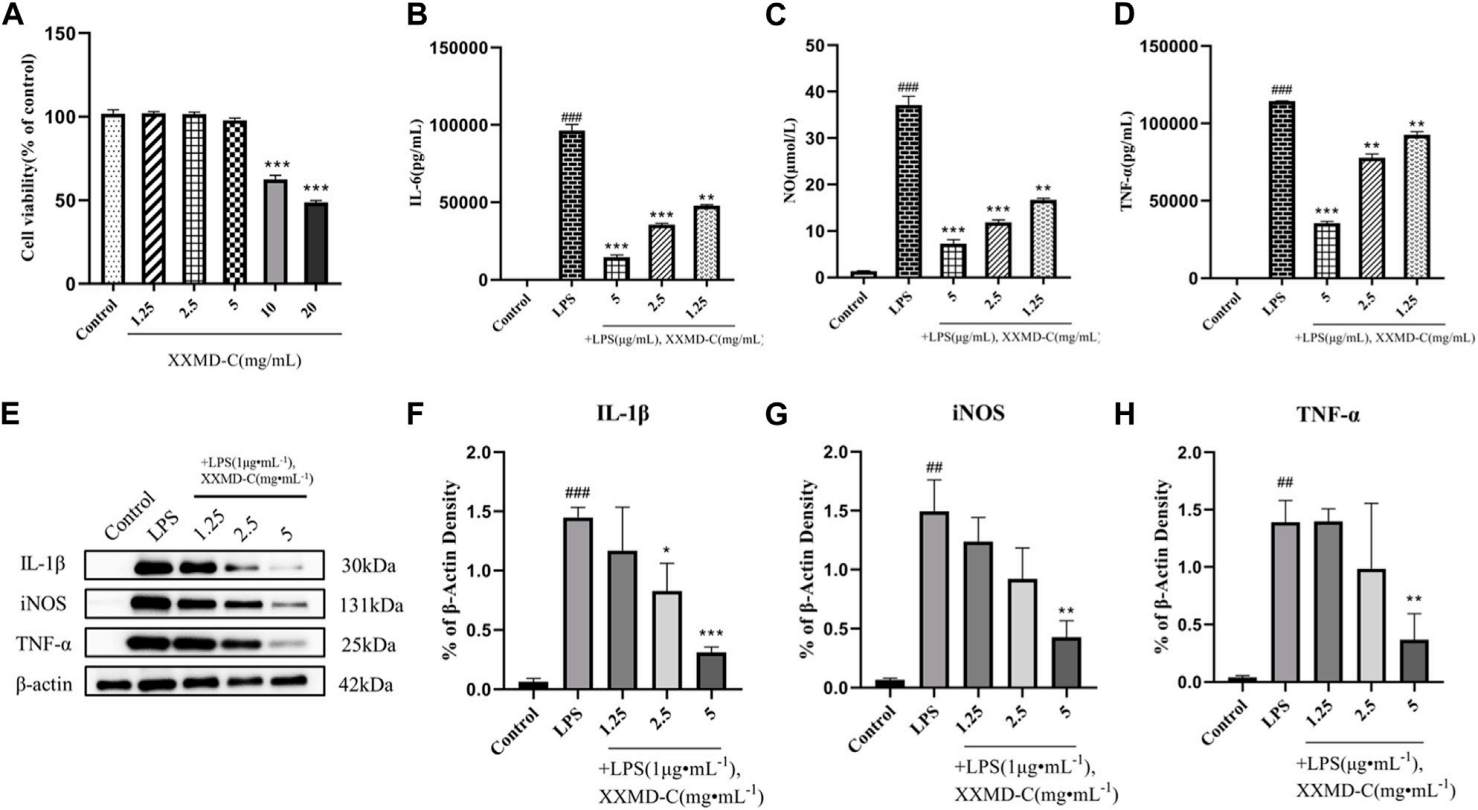
Anti-inflammatory effects of XXMD-C *in vitro*. **(A)** Effect of XXMD-C on the viability of BV-2 cells, *n* = 3.; ELISA for IL-6 **(B)**, NO **(C)** and TNF-α **(D)** expression, *n* = 3; **(E,F,G and H)** Western blot for IL-1β **(F)**, NO **(G)** and TNF-α **(H)** protein expression, *n* = 3. Compared with Control, ^#^ *p* < 0.05, ^##^ *p* < 0.01; compared with LPS, *p < 0.05, **p < 0.01.

The CCK8 assay was used to study the changes in proliferation activity of BV-2 cells treated with different concentrations of XXMD-C. As shown in Figure (Figure 2A), significant changes in cell viability were observed after treatment with different concentrations of XXMD-C. Based on the experimental results, a concentration of 5 mg/mL of XXMD-C was determined to have minimal impact on cell viability. Therefore, XXMD-C at a concentration of 5 mg/mL was selected as the high-dose group, and 2.5 mg/mL and 1.25 mg/mL were chosen as the medium and low-dose groups, respectively, to evaluate the anti-inflammatory effect of XXMD-C on BV-2 cells.

### 3.3 The effects of XXMD-C on NO, IL-6, and TNF-α levels in LPS-induced BV-2 cells

LPS was used to induce an inflammatory model in BV-2 cells. The content of inflammatory factors NO, IL-6, and TNF-α in the cell culture supernatant was measured by ELISA. After LPS treatment, the levels of NO, IL-6, and TNF-α in the cell culture supernatant were significantly increased compared to the normal control group (*p* < 0.001), indicating that LPS can successfully induce the formation of an inflammatory model in BV-2 cells. The NO, IL-6, and TNF-α levels in the cell culture supernatant of different concentrations of XXMD-C were lower than those in the LPS group and showed a dose-dependent effect. Among them, the high-dose group of XXMD-C (5 mg/mL) showed the most significant reduction in inflammation (*p* < 0.001), as shown in the figure (Figures 2B–D). These results suggest that XXMD-C does have anti-inflammatory abilities in the LPS-induced BV-2 cell model and the anti-inflammatory effect is significant.

### 3.4 Western blot analysis of the effects of XXMD-C on NO, IL-1β, and TNF-α levels

LPS-induced BV-2 cell model was extracted and the total protein of the treated cells with XXMD-C was analyzed by Western blot to investigate the effect of XXMD-C on the expression of inflammatory proteins NO, IL-1β, and TNF-α induced by LPS. As shown in Figure (Figures 2E–H), the levels of inflammatory proteins NO, IL-1β, and TNF-α were significantly increased in the BV-2 cell model. However, after treatment with XXMD-C, the levels of inflammatory protein were significantly decreased, indicating that XXMD-C can effectively reduce the inflammatory response in the BV-2 cell model. Further investigation is needed to explore the mechanism of XXMD-C in treating inflammation in BV-2 cells.

### 3.5 Identification of extracellular vesicles derived from BV-2 cells

**FIGURE 3:**
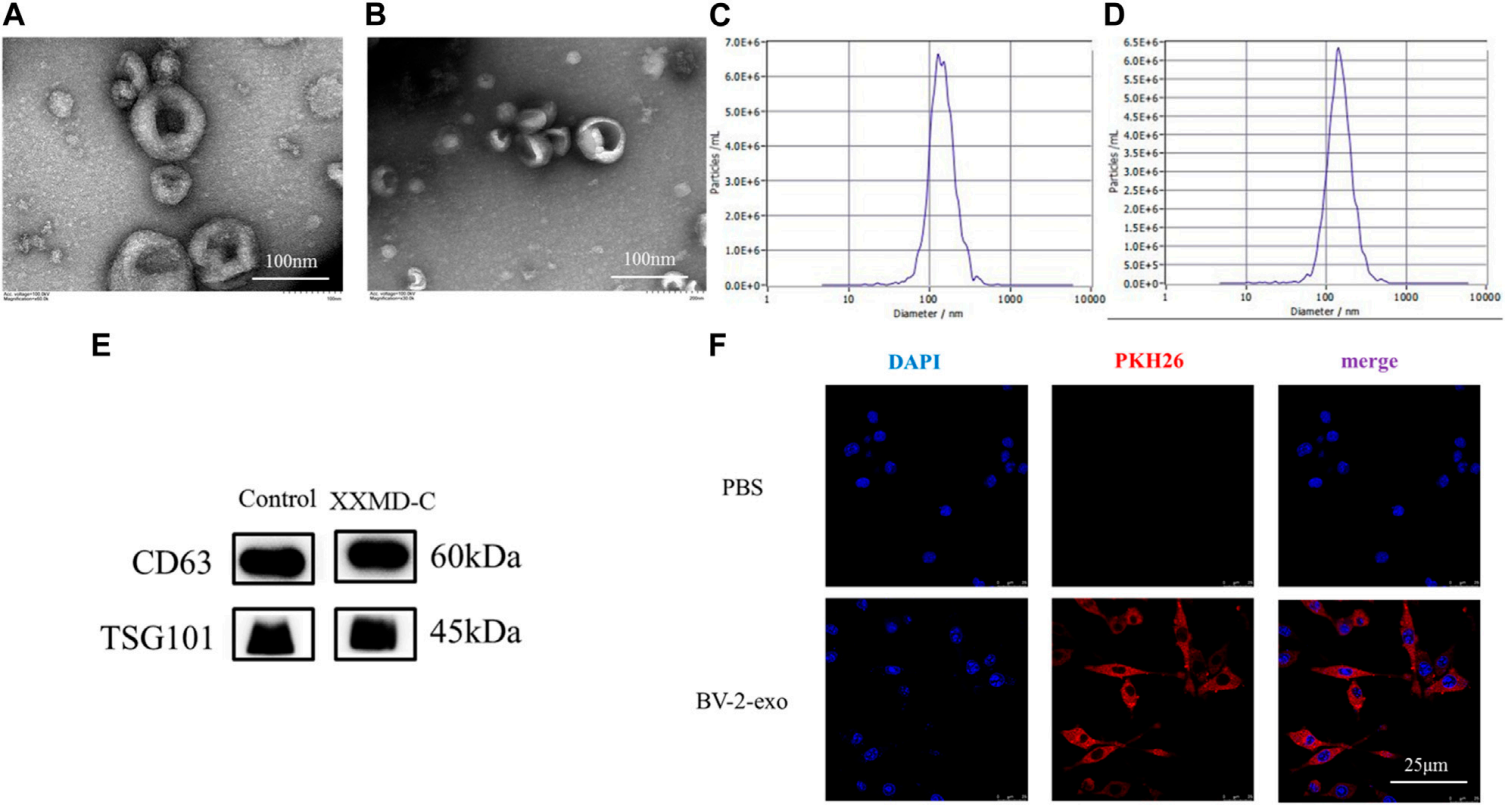
Exosome identification and tracing. Representative transmission electron microscopy images of microglia-derived exosomes **(A)** and microglia-derived exosomes after stimulation with XXMD-C and LPS **(B)**, scale bar 100 nm; nanoparticle size images of microglia-derived exosomes **(C)** and microglia-derived exosomes after stimulation with XXMD-C and LPS **(D)**; **(E)** microglia-derived exosomes were identified by Western blotting labelled as CD63 and TSG101; **(F)** exosome uptake. Microglia DAPI (blue) staining, exosome PKH26 (red) staining, scale bar 25 μm.

The extracted BV-2 exosomes were subjected to nanoparticle tracking analysis, TEM, and protein immunoblotting to detect their size, shape, and specific proteins. The results showed that the exosome size was around 100–150 nm (Figures 3C,D), and the exosomes had a typical cup-shaped or round morphology and a clear lipid bilayer structure under transmission electron microscopy (Figures 3A,B). Immunoblotting showed that the exosome marker proteins CD63 and TSG101 were both positive (Figure 3E). These results indicated that the exosomes extracted were consistent with the characteristics described in the literature in terms of their shape, size, and specific proteins. The successful preparation of exosomes using the ultracentrifugation method enables further mechanistic studies.

### 3.6 Confocal laser scanning microscopy to determine microglial uptake of extracellular vesicles

To determine whether extracellular vesicles (exo) derived from BV-2 cells could be taken up by another BV-2 cell, exosomes were isolated using ultracentrifugation and labeled with PKH26 dye before being added to the small glial cells to assess exosome internalization. As shown in Figure (Figure 3F), immunofluorescence of small glial cells demonstrated DAPI (blue) and exosomes (red), with laser confocal microscopy revealing that the exosomes (red) were absorbed by the cells and located around the nuclei (blue). No red fluorescence signal was detected in the PBS control group. The results showed that exosomes derived from small glial cells were internalized by small glial cells, suggesting that information transfer between small glial cells could occur through exosomes.

### 3.7 Extracellular vesicle small RNA sequencing analysis

#### 3.7.1 Exosomal miRNA profiling

**FIGURE 4:**
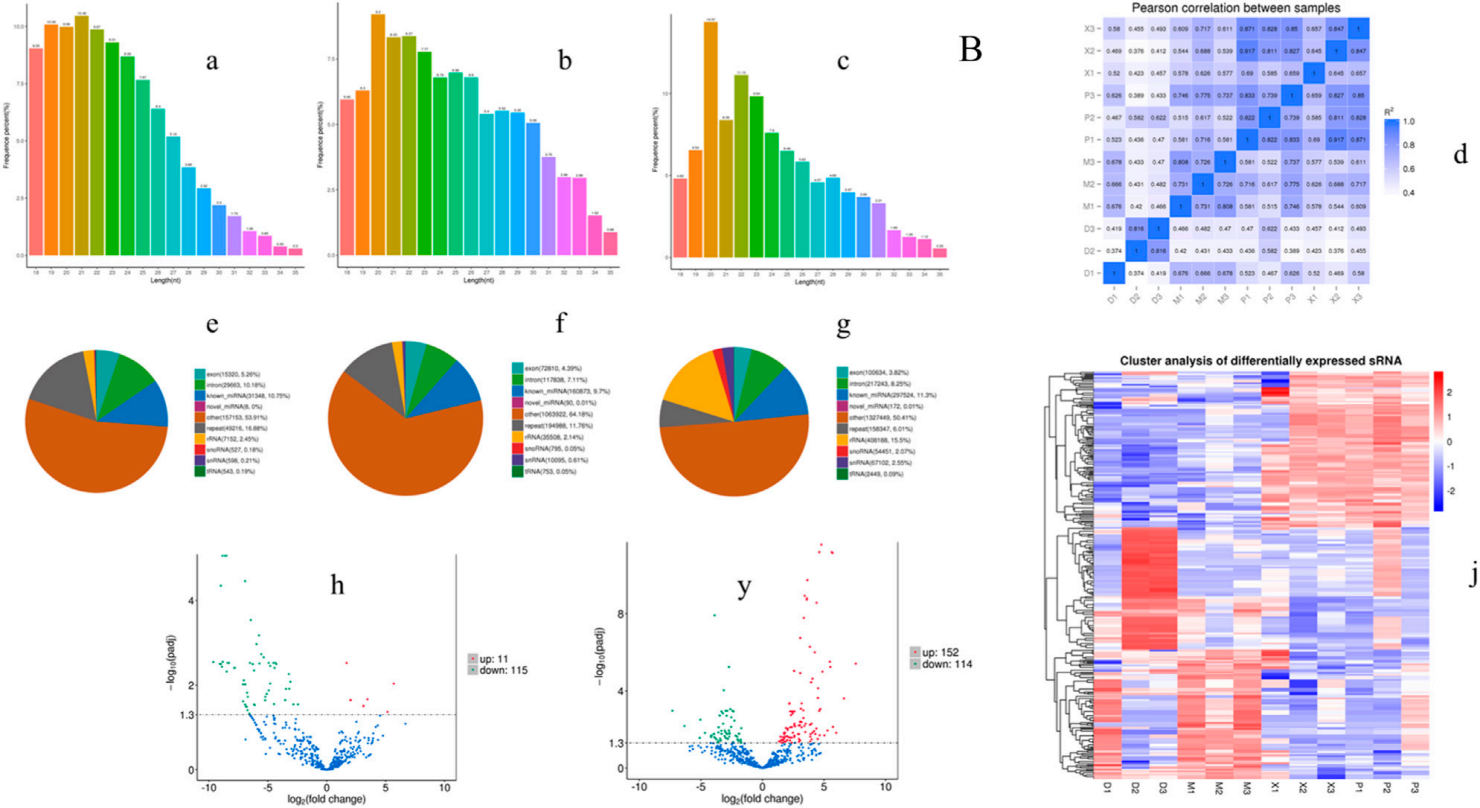
Exosome transcriptome sequencing results. Length distribution statistics of total sRNA fragments obtained in Control group **(A)**, LPS group **(B)** and XXMD-C group **(C)**; **(D)** schematic diagram of miRNA expression correlation between samples; sRNA classification statistics in Control group **(E)**, LPS group **(F)** and XXMD-C group **(G)**; Control group and LPS group **(H)**, XXMD-C group and LPS group (y) Differential miRNA volcano plots between groups, horizontal coordinates indicate the expression fold change (log2FoldChange) of miRNAs between different samples or comparative combinations, vertical coordinates indicate the significance level of expression differences; horizontal dashed lines in the plots correspond to qvalue (default) or *p*-value = 0.05 significance difference threshold, expression upregulated miRNAs are indicated by red dots, downregulated miRNAs are indicated by green dots, and blue dots are miRNAs that did not undergo significant changes; **(J)** clustering plot of differential miRNAs, the upper panel shows the overall hierarchical clustering plot with log10 (TPM+1) values, red indicates high expression miRNAs and blue indicates low expression miRNAs, *n* = 3.

In order to investigate the anti-inflammatory mechanism of XXND-C in BV-2 cells, we performed transcriptome sequencing on extracellular vesicles (EVs) from each group of BV-2 cells. After filtering out reads containing adapters or low-quality bases, the sequencing base quality was mostly Q30, and high-quality small RNA tag sequences that met the relevant requirements were obtained. After removing rRNA and other ncRNAs, the clean small RNA tag sequences were aligned to the reference genome to calculate the alignment rate for subsequent analysis. The gene expression levels between samples were evaluated for correlation, and the expression patterns among the samples were found to be similar (Figure 4D). By comparing and identifying with public databases, the Control group, LPS group, and XXND-C group accounted for approximately 10.75%, 9.70%, and 11.30% of miRNAs(Figures 4E–G), respectively. Generally, the length range of animal sRNAs is 18–35 nt, and miRNAs are concentrated at 21-22 nt (Figures 4A–C). The most significant feature of sRNAs obtained by small RNA sequencing is that a major peak appears at 22bp. These parameters are consistent with the length distribution characteristics of animal samples. By analyzing the major components of miRNAs, cluster heatmap analysis was performed to evaluate the expression patterns of miRNAs (Figure 4J), and differential analysis of miRNA sequence data was conducted between the Control group and LPS group, and between the XXND-C group and LPS group. The volcano plot showed significantly differentially expressed miRNAs (Figures 4H,Y). A total of 126 significantly dysregulated miRNAs, including 11 upregulated and 115 downregulated miRNAs, were detected in the Control group compared to the LPS group. In the XXND-C group compared to the LPS group, 266 significantly dysregulated miRNAs, including 152 upregulated and 114 downregulated miRNAs, were detected.

#### 3.7.2 Differential miRNA screening

**FIGURE 5:**
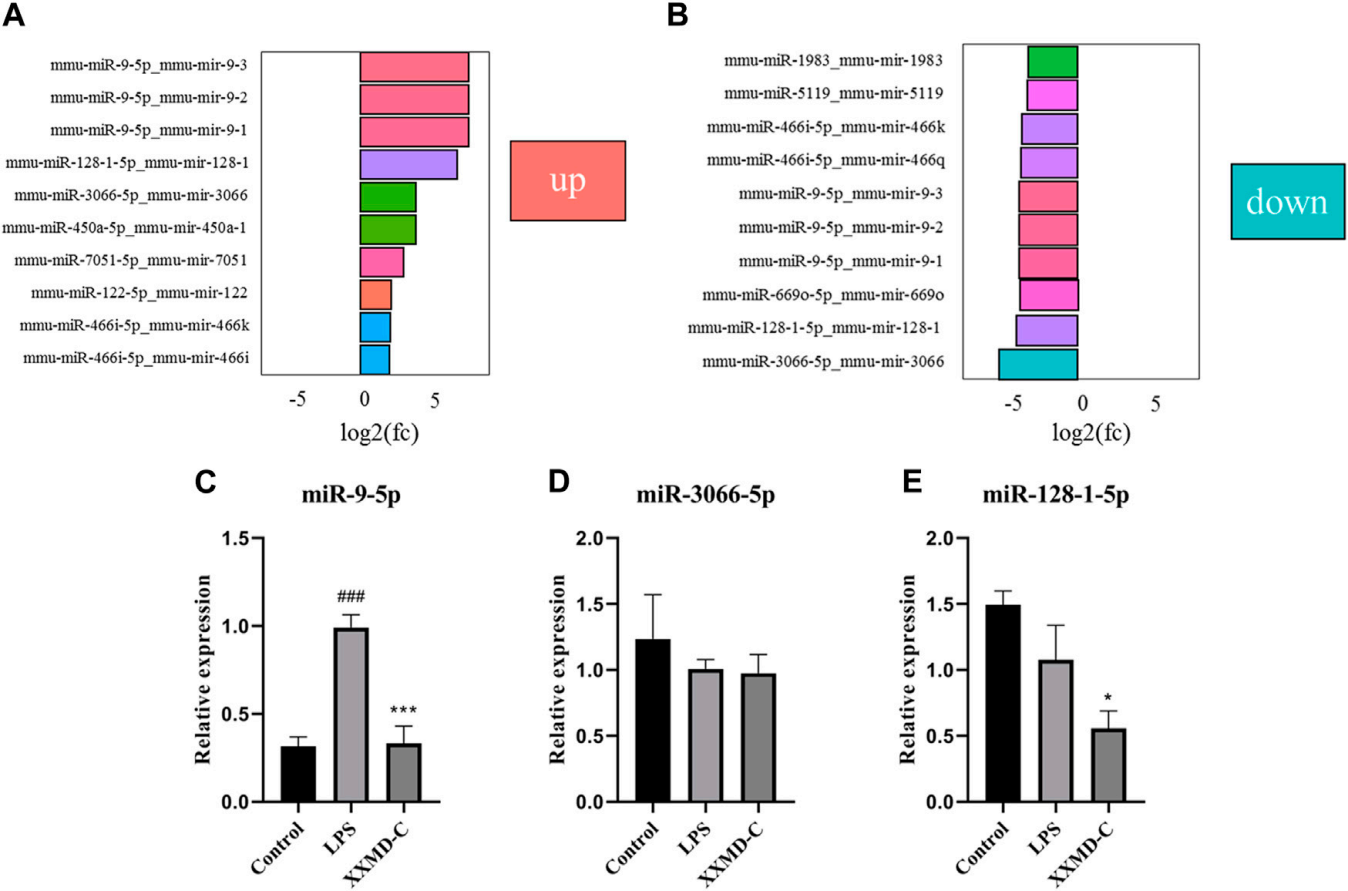
The relative expression of the miR-9-5p, miR-3066-5p and miR-128-1-5p. **(A)** Control and LPS differential miRNA expression histogram; **(B)** XXMD-C and LPS differential miRNA expression histogram; **(C–E)** RT-PCR detection of miR-9-5p, miR-3066-5p, miR-128-1-5p content expression in each group of exosomes, *n* = 3. Compared with Control, ^###^ *p* < 0.001; compared with LPS, ^***^ *p* < 0.001.

The criteria for screening differentially expressed miRNAs are as follows: fold change >1.0, *p* < 0.05, and |log2 (fold change)|>1. Based on the screening criteria, the top 10 differentially expressed miRNAs in the LPS group and the top 10 differentially expressed miRNAs in the XXMD-C group were selected for analysis. It was found that miR-9-5p, miR-3066-5p, and miR-128-1-5p were significantly upregulated in the LPS group, while these three miRNAs were significantly downregulated in the XXMD-C group (Figures 5A,B). Therefore, miR-9-5p, miR-3066-5p, and miR-128-1-5p were selected as our target miRNAs.RT-PCR was used to detect the levels of miR-9-5p, miR-3066-5p, and miR-128-1-5p in extracellular vesicles of each group. The results showed that there was no significant difference in the expression of miR-3066-5p and miR-128-1-5p between the Control group and the LPS group (*p* > 0.05) (Figures 5D,E). Compared with the Control group, the expression of miR-9-5p was significantly increased in the LPS group (*p* < 0.001), while compared with the LPS group, the expression of miR-9-5p was significantly decreased in the XXMD-C group (*p* < 0.01) (Figures 5C–E).

### 3.8 XXMD-C attenuates LPS-induced BV-2 cell inflammatory response by downregulating miR-9-5p

**FIGURE 6:**
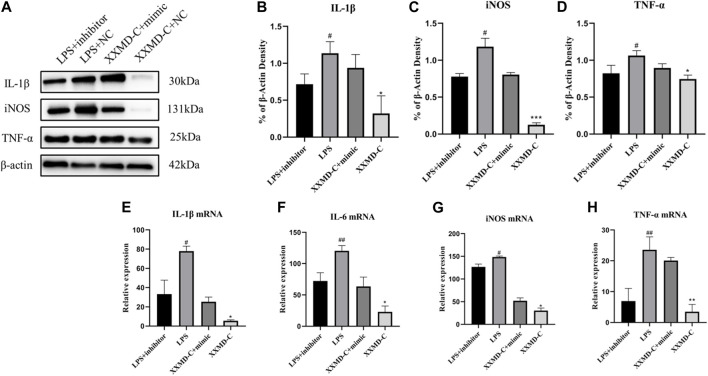
miR-9-5p promotes the development of inflammation. **(A)**Western blot for IL-1β **(B)**, NO **(C)** and TNF-α **(D)** protein expression and qRT-PCR for relative expression of IL-1β **(E)**, IL-6 **(F)**, NO **(G)** and TNF-α **(H)** mRNA., *n* = 3. Compared with LPS + inhibitor, ^#^ *p* < 0.05, ^##^ *p* < 0.01; compared with XXMD-C + mimic, *p < 0.05, **p < 0.01.

miRNAs are a class of small RNA molecules composed of 18–24 nucleotides with a stem-loop secondary structure. They can affect the development of diseases by inhibiting their expression. The preliminary results of this experiment showed that miR-9-5p may be involved in the development of inflammation in BV-2 cells. To further explore the molecular mechanism of miR-9-5p in the process of cell inflammation, NC-inhibitor/miR-9-5p-inhibitor and NC-mimic/miR-9-5p-mimic were transfected into BV-2 cells, and the mRNA expression of inflammatory factors NO, IL-1β, and TNF-α was detected by RT-PCR. The RT-PCR results showed that the expression of NO, IL-1β, and TNF-α was upregulated in BV-2 cells after LPS treatment, downregulated after miR-9-5p inhibitor treatment, and downregulated after XXMD-C treatment. However, this downregulation was reversed after miR-9-5p mimic treatment, as shown in the figure (Figures 6B–D). Western blot analysis of the levels of NO, IL-1β, and TNF-α in each group of microglial cells showed that the levels of inflammatory factors decreased in the LPS group after miR-9-5p inhibitor treatment, while they increased in the XXMD-C group after miR-9-5p mimic treatment. The results are shown in the figure (Figures 6E–H). These results indicate that the expression of miR-9-5p can promote the occurrence of inflammatory response, and XXMD-C regulates the inflammatory response by affecting the expression of miR-9-5p in BV-2 cell-derived extracellular vesicles.

## 4 Disscussion

After cerebral ischemia, the inflammatory process plays an important role in subsequent brain damage. Many clinical trials and basic research studies have shown that the activation of microglia mediates the inflammatory response to a series of brain injuries following stroke (Lo et al., 2005; Chamorro et al., 2016; Shi et al., 2019). In a resting state, microglia constantly interact with other cells and perform the function of monitoring neural homeostasis and responding to injury and infection (Leng and Edison, 2021). Under conditions of abnormal and dangerous stimuli, the inflammatory response in the central nervous system is mainly mediated by activated microglia (Nguyen et al., 2022). As nanoscale cellular vesicles, extracellular vesicles can rapidly complete information transfer between cells, and microglia can secrete extracellular vesicles to propagate inflammation through miRNAs and influence the occurrence and development of neuroinflammation. LPS is an important component of the cell wall of Gram-negative bacteria, and is a strong stimulus for microglial activation (Nam et al., 2018). LPS can increase the release of microglial extracellular vesicles and induce microglial secretion of pro-inflammatory mediators. When the extracellular vesicles secreted by activated microglia are internalized by resting microglia, the miRNAs on the vesicles can rapidly activate the resting microglia and then act on the next microglia by secreting extracellular vesicles. Extracellular vesicles can rapidly spread inflammation between cells, and activate nearby cells in the neurovascular unit of the brain by being secreted by brain endothelial cells that have been activated by systemic inflammation (Holm et al., 2018). Therefore, in this study, LPS was used to induce an inflammatory response in microglia as a model of inflammation, and a new model of microglia “self-interaction dialogue” was established by co-culturing microglia-derived extracellular vesicles with microglia, to investigate the mechanism by which LPS-stimulated microglia spread inflammation via extracellular vesicles.

When BV-2 cells are stimulated with LPS, they quickly activate and release various pro-inflammatory cytokines, including interleukin IL-1β, IL-6, tumor necrosis factor-α (TNF-α), nitric oxide (NO), etc., which affect the occurrence of neuroinflammation by promoting the upregulation of pro-inflammatory factors. Neuroinflammation plays a crucial pathological role in subsequent brain damage after stroke, and IL-1β has been identified as a key cytokine in stroke (Voet et al., 2019). It is a hallmark of early inflammation and can induce local inflammation and the recruitment and activation of neutrophils, monocytes, and macrophages (Chan and Schroder, 2020). IL-6 is considered an inflammatory cytokine that has been reported to cause death of cerebellar granule neurons (Ishijima and Nakajima, 2021). However, IL-6 is a multifunctional cytokine, and in many experiments, it has been shown to have both anti-inflammatory and pro-inflammatory effects. In this study, we consider IL-6 as an inflammatory cytokine. TNF-α is a major Th1-type proinflammatory cytokine and a central mediator of neuroinflammation. In the central nervous system, activated microglia are one of the major sources of TNF-α (Brás et al., 2020). iNOS is usually not expressed in microglia in healthy brains, but inflammatory stimuli such as LPS and cytokines can induce its expression in microglia, leading to the production and release of large amounts of NO following ischemia, trauma, neurotoxicity, or inflammatory injury, which can trigger some pathological processes (Dai et al., 2011). These inflammatory factors are stimulated by external factors, interact with each other, and jointly manage the body’s inflammatory response, participating in the disease process (Zhou et al., 2021).

In our study, we found that high concentrations of XXMD-C significantly decreased the expression of IL-1β, IL-6, TNF-α, and NO, indicating that XXMD-C mediates the expression of inflammatory factors and has a significant anti-inflammatory effect. XXMD-C is composed of four Chinese medicinal materials: Ma Huang, Gui Zhi, Huang Qin, and Ren Shen. According to the 2020 edition of the Chinese Pharmacopoeia, the main components of Ma Huang, Gui Zhi, Huang Qin, and Ren Shen are ephedrine, pseudoephedrine, baicalin, ginsenoside Rg1, ginsenoside Re, and ginsenoside Rb1. This study also detected components in XXMD-C, including ephedrine, pseudoephedrine, cinnamaldehyde, baicalin, baicalein, wogonin, and ginsenoside Rg1, which can represent the four Chinese medicinal materials in XXMD-C. Many components in XXMD-C, such as ephedrine (Zheng et al., 2012; Wu et al., 2014; Wei et al., 2019; Sioud et al., 2020), pseudoephedrine (Li et al., 2019), cinnamaldehyde (Liu et al., 2019; Kim et al., 2020; Gulec Peker and Kaltalioglu, 2021), baicalin (Li et al., 2020; Fu et al., 2021; Yan et al., 2021), and ginsenoside Rg1 (Chen et al., 2022; Zhang et al., 2022), have been reported in relevant literature to have significant anti-inflammatory effects. Therefore, it can be seen that XXMD-C indeed has anti-inflammatory potential.

Transcriptomics is a powerful tool for studying the effects of traditional Chinese medicine formulas on the entire gene expression. It can elucidate complex biological processes and their underlying relationships, allowing us to conveniently and reliably understand the changes in genome-wide RNA expression, thus revealing the multiple mechanisms of drugs (Liu et al., 2015). In this study, we investigated the anti-inflammatory mechanism of XXMD-C through extracellular vesicle transcriptomics. Through extracellular vesicle sequencing analysis, we identified differentially expressed miR-9-5p as our target gene. To confirm miR-9-5p as our target gene, we performed Western blot and RT-PCR experiments. Our study found that inhibiting the expression of miR-9-5p (miR-9-5p inhibitor) led to a decrease in the expression of inflammatory factors IL-1β, IL-6, iNOS, and TNF-α, while increasing the expression of miR-9-5p (miR-9-5p mimic) resulted in an increase in inflammatory factor levels. However, treatment with XXMD-C reversed the effect of miR-9-5p mimic, resulting in a decrease in the levels of inflammatory factors. By reviewing relevant literature, it has been shown that LPS stimulation can induce the expression of miR-9-5p (Akerblom et al., 2013), which in turn can induce polarization of microglia (Yao et al., 2014) and strongly promote the release of inflammatory cytokines (Xian et al., 2022). miR-9-5p can also suppress pro-inflammatory mechanisms by mediating SIRT1 (Ma et al., 2021) and has therapeutic effects on neurotoxicity and inflammation in Parkinson’s disease (Wang et al., 2019). BNIP3, as a target gene of miR-9-5p, can cause hypoxia-induced H9c2 cell damage by upregulating BNIP3 expression. Suppression of miR-9-5p can reduce hypoxia-induced cell viability inhibition, promote cell apoptosis, and inflammation (Cai et al., 2021). Additionally, inhibition of miR-9-5p can induce overexpression of KLF4, which promotes chondrocyte proliferation and migration, while inhibiting cell apoptosis and inflammation (Huang et al., 2021b). The above results indicate that the expression of miR-9-5p can promote inflammation, and XXMD-C has a strong inhibitory effect on the upregulation of miR-9-5p in BV-2 cells induced by LPS stimulation.

Finally, our study has some limitations. Using transcriptome analysis, we only screened for differentially expressed miRNAs and did not further investigate their downstream targets and related pathways, nor did we analyze their biological processes. Additionally, this study was limited to *in vitro* experiments and did not explore whether XXMD-C has the same therapeutic effect in animal models. Further research is needed to determine whether XXMD-C can achieve its anti-inflammatory effect by suppressing miR-9-5p expression. Knocking out the miR-9-5p gene in mice and establishing a stroke model after ischemia in mice to see if inflammation can be treated would also require further exploration and research.

## 5 Conclusion

Based on the above, our *in vitro* studies have demonstrated that XXMD-C exerts an anti-inflammatory effect by inhibiting the expression of relevant inflammatory factors through the suppression of miR-9-5p in extracellular vesicles from microglia. This study identified differential miRNA expression through smallRNA-seq analysis of extracellular vesicles and investigated the mechanism of XXMD-C in treating inflammation after stroke, providing scientific reference for future research on XXMD-C and laying a foundation for its clinical application.

## Data Availability

The authors acknowledge that the data presented in this study must be deposited and made publicly available in an acceptable repository, prior to publication. Frontiers cannot accept a manuscript that does not adhere to our open data policies.
